# Immunobiotics Beneficially Modulate TLR4 Signaling Triggered by Lipopolysaccharide and Reduce Hepatic Steatosis In Vitro

**DOI:** 10.1155/2019/3876896

**Published:** 2019-03-14

**Authors:** Paulraj Kanmani, AbuZar Ansari, Julio Villena, Hojun Kim

**Affiliations:** ^1^Department of Rehabilitation Medicine of Korean Medicine, Dongguk University, Goyang, Republic of Korea; ^2^Laboratory of Immunobiotechnology, Reference Centre for Lactobacilli (CERELA-CONICET), Tucuman, Argentina

## Abstract

Hepatic inflammation and injury may result from the translocation of pathological bacteria and their proinflammatory mediators. Probiotics attenuate hepatic diseases related to inflammation by exhibiting immunoregulatory effects. Therefore, this study was conducted to evaluate lipid reduction and immunoregulatory potentials of probiotic bacteria in vitro. HepG2 cells treated with total cellular fluid (TCF) of LABs reduced lipid accumulation. Moreover, cells responded to lipopolysaccharide (LPS) by producing higher levels of IL-6, IL-8, MCP-1, and TNF-*α*. TCF of LABs treatment showed remarkably diminished levels of the expression of these cytokines via modulation of the expression of TLR-negative regulators, as well as MAPK and NF-*κ*B pathways. Moreover, heat-killed LABs were able to diminish TGF-*β*, IL-1*β*, and IL-6 and to increase IL-10 and TLR4 levels in THP-1 cells. LABs also decreased the protein level of TNF-*α*. These results demonstrated that immunobiotics exhibit potent immunoregulatory activity and may be used as effective therapeutic agents to alleviate inflammatory response.

## 1. Introduction

Because of their various functional capabilities, probiotics and their applications in real-world food and feed industries, as well as in clinical and medical fields, have increased over the last two decades. Probiotics are live beneficial microorganisms that confer health-promoting effects to the host when consumed in adequate amounts [1]. Most probiotics are lactic acid bacteria (LABs), which are a group of bacterial strains primarily found in fermented products and the guts of mammals. *Lactobacillus* sp. and *Bifidobacteria* are promising LABs that are increasingly recognized and utilized by humans as probiotic bacteria. A bacterial strain that has potent immunoregulatory functions is defined as immunobiotic [2]. Accordingly, the potential health-promoting effects of these strains have been extensively studied for use in the treatment of various diseases, including liver diseases. The results of these investigations have shown that these bacteria can withstand gastrointestinal conditions and exert several beneficial effects, including immunomodulation, anti-obesity, strengthening of gut barrier function, anticancer, promotion of dietary uptake, prevention of intestinal inflammation, and increasing antagonism against invaders [3–6]. Several mechanisms have been postulated to be responsible for these beneficial effects of probiotics; however, they are mainly achieved through the regulation of the intestinal immune system and maintenance of gut microflora homeostasis [7].

The liver is a primary regulator of several physiological processes and many diseases, including liver inflammation, hepatic steatosis, fibrosis, cirrhosis, hepatocellular carcinoma, and hepatitis that are known to affect liver functions [8]. However, the definite pathological mechanisms responsible for the development of chronic liver diseases are not yet known. Lipopolysaccharide (LPS) is an important cell wall component of Gram-negative bacteria that has been found to induce inflammatory response in infected cells and lead to sepsis or endoxima and organ failure [8, 9]. Increased circulation of bacterial LPS has been reported to stimulate hepatocyte cells to produce higher amounts of proinflammatory cytokines and fibrogenesis markers that induce liver inflammation and progression of liver diseases [10, 11]. A significantly higher level of LPS was found in patients with liver diseases [12, 13], indicating that the liver is not simply a primary regulator of physiological functions but also a preeminent site or organ for the clearance of bacterial components and its toxicity [14]. Lipopolysaccharide has been shown to induce production of reactive oxygen species (ROS) and proinflammatory cytokines/chemokines by hepatic cells through interaction with pattern recognition receptors (PPRs) such as Toll-like receptors (TLRs) [10, 15].

Toll-like receptor 4 (TLR4) is an active receptor for LPS. Once it recognizes LPS, TLR4 promptly mediates inflammatory signaling, which results in increased production of different inflammatory cytokines/chemokines in the inflamed site [15]. TLR4 can be expressed by most liver cell types and actively responds to LPS by producing an array of proinflammatory cytokines in vivo [10]. Many studies have confirmed the pathogenic role of TLR4/LPS signaling in the development of alcoholic and nonalcoholic liver diseases, hepatocellular carcinoma, hepatitis B and C, and primary biliary cirrhosis [15]. An in vivo study showed that LPS signaling induced hepatic fibrogenesis in a mouse model that was found to be less susceptible to the development of hepatic fibrogenesis when deficient for TLR4 [16]. TLR4/LPS signaling induces hepatic fibrogenesis through the activation of Kupffer and Hepatic stellate cells to produce higher levels of profibrogenic cytokines (TGF-*β*), chemokines (CCL2, CCL3, and CCL4), and adhesion molecules (ICAM-1 and VCAM-1) [10].

Alcoholic and nonalcoholic liver diseases are closely associated with changes in gut microbiota composition. Administration of probiotics has been shown to ameliorate the progression of liver diseases by modulating the host bacterial community [17, 18]. In addition, probiotics were able to suppress LPS-induced hepatic injury in a mouse model by maintaining intestinal barrier function [19]. Probiotics may cause these positive intestinal and hepatic effects via different mechanisms that include providing substrates for short-chain fatty acid (SCFA) production and facilitating the bioavailability of bile acids [20]. Another in vivo study showed that treatment with probiotics (VSL#3) improved the development of liver steatosis in ob/ob mice [21]. Moreover, the clinical importance of these (VSL#3) probiotic strains was confirmed in a cohort of patients with liver diseases [22]. These studies confirmed the beneficial activity of probiotics in the prevention of liver diseases in vivo. Indeed, the beneficial effects of probiotics are strain-specific and their functional properties; therefore, the screening and selection of most active strains with potent probiotic effects are very important to achieving target therapeutic effects. Most studies have used in vivo models to evaluate the probiotic effects, while there have been fewer attempts in vitro, and no clear mechanisms explored for the probiotic actions in vivo and in vitro have been identified. In our previous study, we used hepatoma HepG2 cells to screen potent probiotic strains and analyze their immunoregulatory effects [23]. In this study, we selected three different lactic acid bacteria based on their antimicrobial activity, reduction of fat accumulation, and bile and acid stability. HepG2 cells were used to screen the most active probiotic strains with antihepatic steatosis and immunomodulatory capabilities, as well as to study the molecular mechanisms involved in the immunoregulatory activity of probiotics.

## 2. Materials and Methods

### 2.1. Cell Culture

Human hepatoma HepG2 cells from the Korean cell line bank (Seoul, South Korea) were purchased and maintained in Dulbecco's modified Eagle's medium (DMEM, Gibco, USA). For the experiments, cells were cultured in DMEM/high-glucose medium (Gibco™) containing 10% fetal bovine serum (FBS, Gibco, USA) and 1% penicillin/streptomycin at 37°C under 5% CO_2_. The media was changed at every two-day interval for 5-6 days. In addition, the human monocytic cell line (THP-1) was purchased from the Korean cell line bank and maintained in RPMI-1640 medium (Gyeongsangbuk-do, South Korea) until used for a coculture investigation.

### 2.2. Bacterial Strains

Three different lactic acid bacteria (LABs) were isolated from Korean fermented foods. The phenotypical and genotypical characterizations were performed with appropriate techniques and identified as *Weissella cibaria* strain DUHJ2, *Lactobacillus sakei*, and *L. plantarum* strain DUHJ2 (hereafter referred to as WCI, LSA, and LPL, respectively). The strains were cultured in MRS broth at 37°C for 19 h, after which they were centrifuged, washed once with DPBS, and subsequently mixed with DMEM at appropriate concentrations, then stored at -4°C until further investigation.

### 2.3. Induction of Hepatic Steatosis (HS) and Differentiation of THP-1 Cells

Hepatic steatosis was induced as described previously [23]. Briefly, hepatoma HepG2 cells were cultured (5 × 10^4^ cells/ml) in 12-well type I collagen-coated plates (SPL Life Sciences Co. Ltd., Gyeonggi-do, South Korea) at 37°C for 3-4 days. The confluent cells were then incubated with DMEM supplemented with 10% fetal bovine serum (FBS), 1% penicillin/streptomycin, 1% bovine serum albumin (BSA), and 1 mM free fatty acid [FFA (2 : 1 of oleic acid (0.66 mM) and palmitic acid (0.33 mM) prepared in 100% isopropanol)] for 24 h. Finally, the incubated cells were washed with distilled phosphate-buffered saline (DPBS) until further analysis.

To differentiate THP-1 cells, they were cultured in RPMI-1640 supplemented with 1% FBS, 1% penicillin/streptomycin, and 0.05 mM mercaptoethanol at 37°C for 5-6 days. The cells were then incubated with differentiation medium (RPMI 1640 media with 50 ng/ml of PMA (Phorbol 12-Myrisate 13 Acetate)) for 48 h to induce conversion into macrophage-like cells. Finally, differentiation medium was replaced with fresh RPMI medium and subsequently incubated at 37°C for 24 h, after which the differentiated cells were used to prepare CFS of LAB strains and to coculture with HepG2 cells.

### 2.4. Preparation of Total Cellular Fluid (TCF) of LAB Strains

The total cellular fluid of LABs was prepared as previously described [23]. Briefly, differentiated THP-1 cells were cultured (1 × 10^6^ cells/well) in 6-well type I collagen-coated plates (SPL Life Sciences Co. Ltd., Gyeonggi-do, South Korea) and incubated with LAB strains (5 × 10^7^ cells/well) in RPMI media. After 24 h of incubation, the total cellular fluids (TCFs) were transferred to fresh tubes, centrifuged, and filter sterilized to remove cellular contents, after which they were stored at -4°C for further stimulation studies. The cytotoxicity of LAB strains and their TCFs was determined in hepatoma HepG2 cells using a cell viability, proliferation, and cytotoxicity assay kit (EZ-CYTOX, DOGEN Bio Co. Ltd.).

### 2.5. Reduction of Hepatic Steatosis

To evaluate the reduction in hepatic steatosis, HepG2 cells were cultured (3 × 10^4^ cells/ml) in 12-well collagen-coated plates at 37°C for 3 days, then incubated with TCF (25 *μ*l and 50 *μ*l/ml) of different LABs for 48 h after treatment with 1 mM of FFA for 24 h. The cells were then washed with DPBS and fixed with formalin (10%) at room temperature (RT) for 5 minutes, after which they were fixed with fresh formalin (10%) at RT for 1 h and subsequently washed with isopropanol (60%). The completely dried plates were then stained with Oil Red O (ORO) solution (0.5%) for 10 minutes at RT, washed four times with deionized water (DW), and observed under a microscope (Leica DMI 6000B, Wetzlar, Germany). To quantify the amount of fat that accumulated in the cells, 1 ml of isopropanol (100%) was added to each well and allowed to stand for 5 minutes at RT, after which absorbance was read at 520 nm using a microplate reader (SpectraMax Plus 384, San Jose, CA, USA).

### 2.6. TCF of LABs Ameliorates LPS-Induced Inflammatory Response in HepG2 Cells

HepG2 cells were cultured (3 × 10^4^ cells/ml) in 12-well plates at 37°C under 5% CO_2_. Once fully confluent, cells were stimulated with 50 *μ*l TCF of LABs for 48 h before and after treatment with FFA for 24 h. The stimulated cells were then washed with DMEM medium and poststimulated with 1 *μ*g/ml of LPS (lipopolysaccharide from *E. coli* O55.B5, Sigma-Aldrich, USA) for 3 and 12 h. Finally, the mRNA levels of proinflammatory cytokines/chemokines (IL-6, IL-8, MCP-1, and TNF-*α*) were analyzed by RT-PCR.

### 2.7. Coculture Study

To determine the immunomodulatory activity of LABs, HepG2 cells (3.5 × 10^4^ cells/well) were cultured in the upper chamber of transwell culture inserts (transparent PTFE membrane-coated collagen, 0.4 *μ*m pore size) (Transwell-COL, Corning Inc., NY, USA) at 37°C under 5% CO_2_. After 5-6 days, the confluent cells were cocultured with macrophage-like THP-1 cells and then cultured in the lower chamber. The apical monolayer of HepG2 cells (TEER value 486 Ω cm^2^) was subsequently stimulated with heat-killed LABs for 48 h, after which 1 *μ*g/ml of LPS was added to the basolateral side and incubation was continued at 37°C for 12 h. The cellular fluid (CF) from both apical and basolateral sides was collected in fresh tubes and stored at -4°C to determine the amount of TNF-*α* production at the protein level using a Human TNF-*α* Quantikine Enzyme-Linked Immunosorbent Assay (ELISA) kit (R&D System, MN, USA) according to the manufacturer's instructions. In addition, RNA from THP-1 cells was extracted by adding TRIzol, after which the expression of cytokines and receptors (IL-10, TGF-*β*, IL-6, IL-1*β*, TLR2, and TLR4) was analyzed by RT-PCR [23].

### 2.8. Quantitative Real-Time Polymerase Chain Reaction (qRT-PCR)

Total RNA from cells was extracted by adding TRIzol. The extracted RNA was used to analyze the expression of different cytokines using RT-PCR as previously described [23]. Primers used in this study were mentioned in Supplementary Table 1.

### 2.9. Modulation of MAPK and NF-*κ*B Pathways by TCF of LABs in HepG2 Cells

Hepatoma HepG2 cells were cultured (1.8 × 10^5^ cells/dish) in dishes (60 mm) at 37°C and 5% CO_2_ for 5-6 days. Fully confluent HepG2 cells were then stimulated with the TCF of LABs at 37°C under 5% CO_2_ for 48 h, then poststimulated with LPS (1 *μ*g/ml) for 0, 10, 30, 60, and 120 min. The cells were subsequently washed with DPBS and lysed by adding 200 *μ*l of CelLytic M cell lysis reagent (Sigma-Aldrich, St. Louis, MO, USA), scraped, and transferred to fresh Eppendorf tubes that were stored at −70°C for blotting. The concentration of protein in the lysed sample was analyzed using a bicinchoninic acid (BCA) assay kit (Thermo Scientific, Pierce, Rockford, IL, USA). Equal amounts of samples were then loaded into 10% SDS-polyacrylamide gels, after which the proteins were separated by electrophoresis at a constant voltage and then transferred onto nitrocellulose membranes (Trans-Blot Turbo™, BioRad). The membranes were subsequently cut at the desired part and incubated with blocking buffer prior to incubation with antibodies to the targeted proteins. The phosphorylation of p38 MAPK and p65 NF-*κ*B degradation were evaluated by incubating the membranes with MAPK Phospho-p38*α* (T180/Y182) antibody (p-p38*α*, Cat. #MAB8691, R&D Systems, MN, USA), NF-*κ*B phospho-p65 (p-p65, Cat. #9242), and *β*-actin antibody (Cat. #4970) (dilution: 1 : 1000) from Cell Signaling Technology (Beverly, MA, USA) overnight at room temperature. Next, membranes were washed with buffer and subsequently incubated with goat anti-rabbit IgG-HRP polyclonal antibody (dilution: 1 : 5000) (AbFrontier, Cat. #LF-SA8002). After 2 h, the membranes were washed with buffer and the protein bands were analyzed by adding a mixture (1 : 1 ratio) of western blot detection solution A and B (SUPEX, Neonex Co. Ltd., Postech, South Korea). Finally, the area of the densitogram peak was estimated using ImageJ software (National Institute of Health, Bethesda, MD, USA).

### 2.10. Modulation of LPS-Induced TLR-Negative Regulator Expression in HepG2 Cells

Both FFA-treated and FFA-untreated HepG2 cells were stimulated with the TCF of LABs at 37°C under 5% CO_2_ for 48 h. After washing with DMEM medium, both cells were poststimulated with 1 *μ*g/ml of LPS for 3 and 12 h. Next, the cells were washed with DPBS, lysed, and scraped by adding a TRIzol reagent, after which the transferred samples were stored at −4°C to extract the RNA. Finally, the mRNA levels of TLR-negative regulators (A20, SIGIRR, Tollip, and IRAKM1) were analyzed by RT-PCR.

### 2.11. Statistical Analysis

Statistical analyses were performed using the SPSS software package (SPSS 12.0, SPSS Inc., Chicago, IL, USA). One-way analysis of variance (ANOVA) was conducted, and the significance of each mean value was determined by Tukey's and Duncan's multiple range tests with a *p* < 0.05 considered to indicate significance.

## 3. Results

### 3.1. LABs Reduce Hepatic Steatosis In Vitro

**Figure 1:**
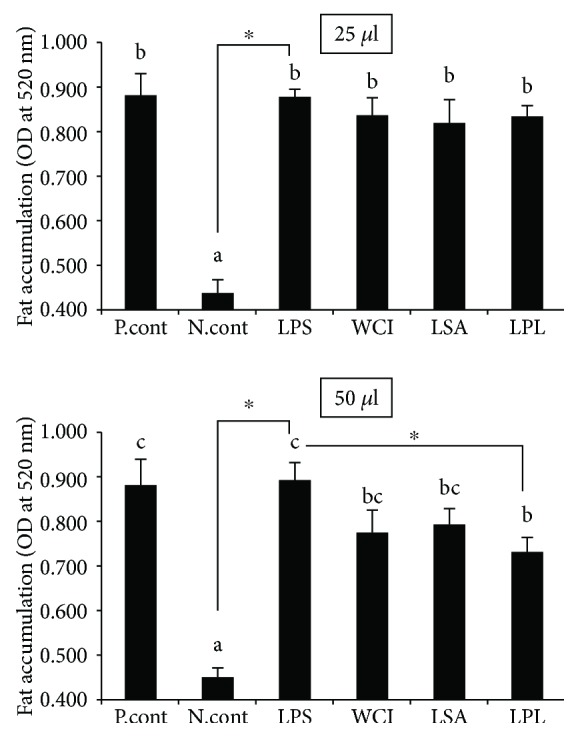
Lipid reduction potential of LABs in vitro. Free fatty acid-treated HepG2 cells were incubated with total cellular fluid of LABs (25 *μ*l and 50 *μ*l/ml) and lipopolysaccharide (LPS) for 48 h, and the amount of lipid reduced by total cellular fluid of LABs in the cells was quantified using a spectrophotometer at 520 nm. Different superscript letters indicate significant differences at the 0.05 level. Asterisks indicate significant differences (*p* < 0.05) between the samples and LPS.

In this study, we used three different LAB strains (*Weissella cibaria* strain DUHJ2, *Lactobacillus sakei*, and *L. plantarum* strain DUHJ2) that were isolated from Korean fermented foods. The isolated strains and their TCF did not affect the growth and proliferation of HepG2 cells and retained cells alive, similar to the control. In addition, we analyzed whether the TCF of LABs decreased the induction of hepatic steatosis in vitro using HepG2 cells. As shown in Figure 1, the TCF of LABs reduced FFA-induced hepatic steatosis by decreasing the accumulation of lipids in HepG2 cells. However, strain- and TCF level-dependent variations were observed in the reductions. Specifically, a higher amount of TCF (50 *μ*l) showed better reduction than lower amounts (25 *μ*l). Among LABs, the TCF of LPL led to a significant decrease in lipid accumulation (more than 18%) compared with LPS, followed by the TCF of WCI and LSA, respectively.

### 3.2. LABs Attenuate LPS-Induced Inflammatory Response in HepG2 Cells

**Figure 2:**
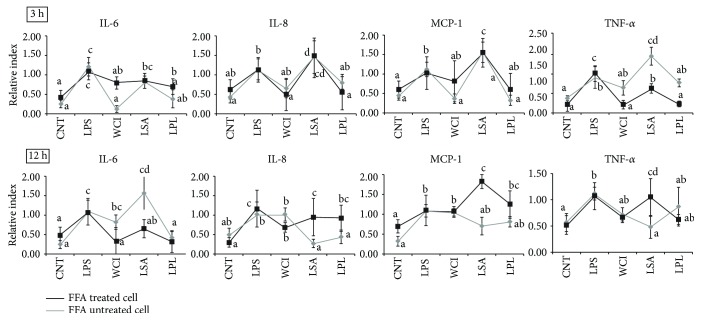
Reduction of inflammatory response induced LPS in HepG2 cells. Both normal and free fatty acid-treated HepG2 cells were stimulated with total cellular fluid (50 *μ*l/ml) of LABs for 48 h, then poststimulated with LPS for 3 h and 12 h. The expression of inflammatory cytokines (IL-6, CXCL8, CCL2, and TNF-*α*) was determined by real-time polymerase chain reaction (RT-PCR). HepG2 cell treated with LPS was used as control. Different superscript letters indicate significant differences at the 0.05 level.

We next analyzed whether the TCF of LABs dampens inflammatory cytokine production in vitro. For this analysis, both FFA-treated and FFA-untreated HepG2 cells were prestimulated with the TCF of LABs, followed by stimulation with LPS for 3 and 12 h. The results of RT-PCR are shown in Figure 2. Treatment with LABs significantly decreased the expression of inflammatory cytokine/chemokines in HepG2 cells. However, these reductions varied with time, cells, and strains. At 3 h, LPS induced the expression of all cytokines/chemokines in both FFA-treated and FFA-untreated HepG2 cells. Moreover, FFA-treated cells stimulated with the TCF of WCI and LPL showed downregulated mRNA levels of IL-6, IL-8, MCP-1, and TNF-*α*, while the TCF of LSA failed to suppress the FFA-treated cells to lower levels of IL-8 and MCP-1. The TCF of LABs exhibited different patterns of inhibition in FFA-untreated cells compared to FFA-treated cells. At 12 h, the TCF of all LABs was able to decrease the expression of IL-6 in FFA-treated cells, but not in FFA-untreated cells (except for LPL). The levels of IL-8 and MCP-1 were also significantly reduced by TCF of all LABs except WCI in FFA-untreated cells, whereas FFA-treated cells responded differentially to the TCF of LABs (Figure 2). Moreover, the TCF of WCI and LPL stimulations decreased the expression of TNF-*α* in both cell types, while the TCF of LSA failed to reduce TNF-*α* in FFA-treated cells. These results indicate that both FFA-treated and FFA-untreated cells were highly responsive to the TCF of LABs, showing different patterns of cytokine expression after stimulation for different times.

### 3.3. LAB Strains Reduce Production of TNF-*α* in a Coculture Model

**Figure 3:**
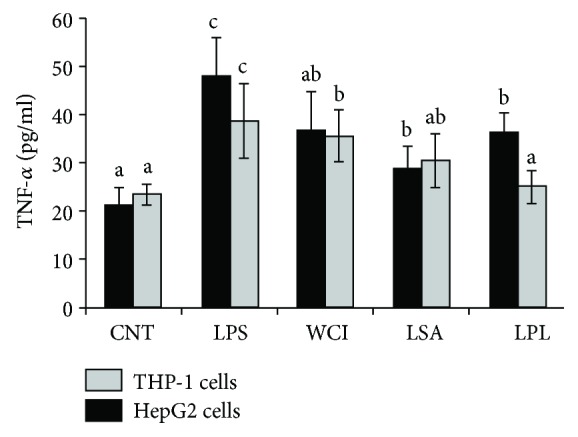
Reduction of TNF-*α* production in HepG2 cells and THP-1 cells. In this coculture study, the apical side HepG2 cells were stimulated with heat-killed LABs for 48 h, after which the basolateral THP-1 cells were treated with LPS for 12 h. The protein levels of TNF-*α* in both apical and basolateral sides were determined. Cell treated with LPS was used as control. Different superscript letters indicate significant differences at the 0.05 level.

To determine if LABs suppress THP-1 cells indirectly through the influence of HepG2 cells in vitro, we cocultured hepatoma HepG2 cells with THP-1 cells and stimulated them with heat-killed LABs followed by LPS treatment for 12 h. As shown in Figure 3, ELISA revealed that all LAB strains were able to suppress THP-1 to produce higher amounts of TNF-*α*. Strain LSA showed better activity than other strains. In contrast, different patterns of TNF-*α* production were observed in HepG2 cells. When compared to THP-1 cells, hepatoma HepG2 cells showed a lower response to LAB strains.

### 3.4. Reduction of mRNA Level of Inflammatory and Anti-Inflammatory Cytokines by LAB Strains in THP-1 Cells

**Figure 4:**
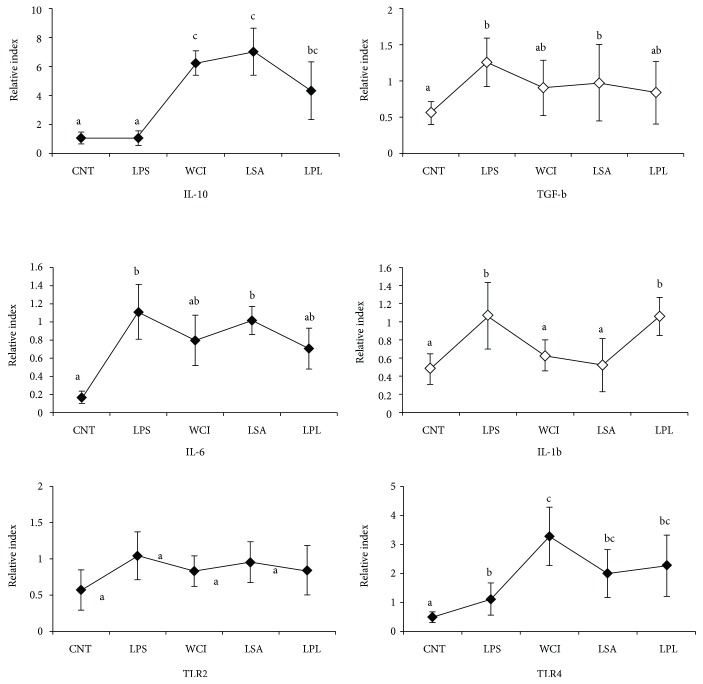
In vitro analysis of anti-inflammatory and inflammatory cytokines in THP-1 cells. In this coculture study, the apical side HepG2 cells were stimulated with heat-killed LABs for 48 h, after which basolateral THP-1 cells were treated with LPS for 12 h. The mRNA levels of IL-10, TGF-*β*, IL-6, IL-1-*β*, TLR2, and TLR4 at mRNA were determined by RT-PCR. Cell treated with LPS alone was used as control. Different superscript letters indicate significant differences at the 0.05 level.

We also investigated whether LAB strains modulate the expression of inflammatory and anti-inflammatory cytokines in THP-1 indirectly through HepG2 cells. The results of RT-PCR showed that stimulation of cells with LABs increased the mRNA level of IL-10, whereas it decreased the expression of TGF-*β* (Figure 4). In addition, LPS induced the expression of IL-6 in THP-1 cells, and all LABs except LSA reduced this LPS-induced IL-6 expression indirectly. The level of IL-1*β* was downregulated by all LABs, but not by LPL. We also analyzed the expression of TLR receptors in THP-1 cells. The expression of TLR2 was not significantly altered by LAB strains, but all LABs showed significantly increased expression of TLR4 in THP-1 cells. These results confirm that isolated LAB strains could suppress cells not only via direct contacts but also through indirect contacts to produce lower levels of inflammatory cytokines in response to LPS.

### 3.5. Modulation of MAPK and NF-*κ*B Pathways

**Figure 5:**
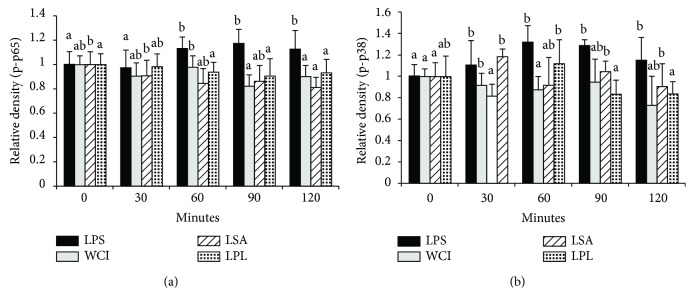
Inhibition of p65 NF-*κ*B and p38 MAPK phosphorylation in HepG2 cells. HepG2 cells were prestimulated with total cellular fluid of LABs for 48 h, then poststimulated with LPS for 0, 30, 60, 90, and 120 min. The phosphorylation of p65 NF-*κ*B and p38 MAPK was determined by western blot analysis. Bar graphs represent the results of three independent experiments, and different superscript letters indicate significant differences at the 0.05 level.

To determine if the TCFs of LABs modulate LPS-induced MAPK and NF-*κ*B pathways in vitro, we stimulated HepG2 cells with the TCF of LABs for 48 h followed by LPS for 0, 30, 60, 90, and 120 minutes. Blotting analysis showed that LABs were able to modulate LPS-induced p65 NF-*κ*B and p38 MAPKs activation in HepG2 cells (Figure 5(a) and 5(b)). However, these modulations varied remarkably in a time-dependent manner. Stimulation of cells with LPS increased the level of p65 NF-*κ*B after stimulation for various lengths of time, but these were reduced when cells were stimulated with the TCF of LABs. The TCF of all LABS remarkably decreased the levels of p-p65 compared to LPS after 30 minutes, and this continued for up to 120 minutes (Figure 5(a), Supplementary Figure 1). Similarly, cells stimulated with LPS increased the phosphorylation of p38 MAPKs in HepG2 cells; however, this LPS-induced p38 MAPK activation was inhibited when cells were treated with the TCF of LABs (Figure 5(b)). Treatment with the TCF of WCI led to reduction at all stimulation points, but this difference relative to LPS was only significant (*p* < 0.05) at 60 minutes. However, the TCF of LSA and LPL diminished the level of p-p38 MAPK at all stimulation points, but significantly lower levels were observed at 30 and 120 minutes, respectively. These results indicate that the TCFs of LABs were able to decrease the expression of inflammatory cytokine/chemokines by modulating the MAPK and NF-*κ*B pathways in HepG2 cells.

### 3.6. Regulation of TLR-Negative Regulator Expression by LABs in HepG2 Cells

**Figure 6:**
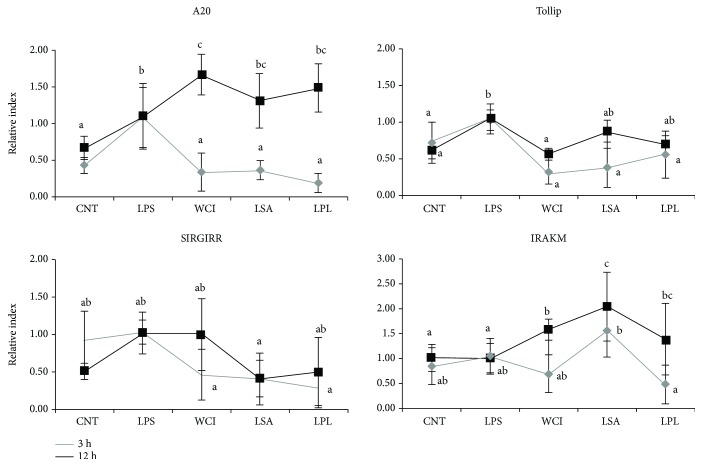
Modulation of the expression of TLR negative regulators by total cellular fluid of LABs in free fatty acid-treated HepG2 cells. HepG2 cells were prestimulated with total cellular fluid of LABs followed by stimulation with LPS for 3 h and 12 h. The expression of A20, SIGIRR, Tollip, and IRAK-M1 was determined by RT-PCR. HepG2 cell treated with LPS was used as control. Different superscript letters indicate significant differences at the 0.05 level.

Several negative regulatory proteins are known to play roles in the regulation of TLR signaling. Therefore, we evaluated whether the TCFs of LABs modulate the expression of TLR-negative regulatory proteins in HepG2 cells under inflammatory conditions. We performed this experiment in both FFA-treated and FFA-untreated HepG2 cells. The expression of TLR-negative regulators in FFA-treated HepG2 cells at 3 and 12 h is shown in Figure 6. Stimulation of FFA-treated cells with LPS increased the expression of negative regulators at all stimulation hours. These LPS-induced expressions were modulated when cells were prestimulated with the TCF of LABs. However, these modulations varied significantly depending on the stimulation hours. The TCF of all LABs decreased the expression of A20 at 3 h, while they increased A20 expression at 12 h compared to LPS (Figure 6). However, the levels of Tollip and SIGIRR were diminished by the TCFs of all LABs at both 3 and 12 h. Conversely, the mRNA level of IRAKM was significantly upregulated at 12 h after stimulation of cells with the TCF of LABs.

**Figure 7:**
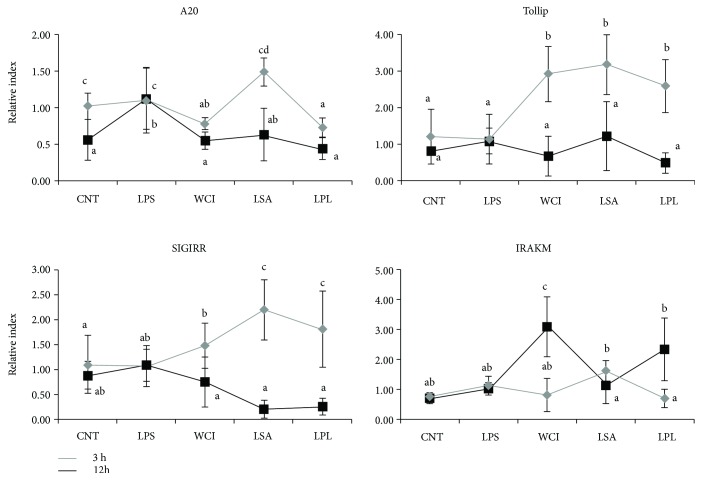
Modulation of TLR-negative regulator expressions by TCF of LABs in HepG2 cells not treated with free fatty acid (normal cells). HepG2 cells were prestimulated with total cellular fluid of LABs and poststimulated with LPS for 3 h and 12 h. The expressions of A20, SIGIRR, Tollip, and IRAK-M1 were determined by RT-PCR. HepG2 cell treated with LPS was used as control. Different superscript letters indicate significant differences at the 0.05 level.

We also analyzed the expression of TLR-negative regulators in FFA-untreated cells. When compared to FFA-treated cells, FFA-untreated cells responded differentially to the TCF of LABs (Figure 7). Stimulation of FFA-untreated cells with the TCF of LABs decreased the expression of A20 at both 3 and 12 h. However, the mRNA levels of Tollip and SIGIRR were increased by the TCF of all LABs at 3 h, while they were decreased at 12 h. Conversely, the TCF of all LABs except LSA showed significantly higher levels of IRAKM than LPS at 12 h, while decreased levels of IRAKM were observed for the TCFs of WCI and LPL at 3 h. These results confirm that the TCFs of LABs were able to regulate TLR signaling by modulation of the expression of TLR-negative regulators in HepG2 cells.

## 4. Discussion

This study shows that the TCFs of LABs were able to diminish hepatic steatosis by reducing the accumulation of lipids, as well as to attenuate bacterial LPS-induced inflammatory response in hepatoma HepG2 cells, which is a well-characterized, transformed cell line model that has been extensively used to analyze the beneficial effects of several components and bacterial strains on hepatic functions at the cellular level in vitro [24–26]. Elevated levels of free fatty acids are thought to be a critical factor of liver diseases such as nonalcoholic liver diseases and hepatic steatosis in obese individuals [27, 28]. Therefore, in this study, we used free fatty acids to induce hepatic steatosis in HepG2 cells to analyze the beneficial effects of LAB TCF in vitro. Cells incubated with free fatty acids showed increased accumulation of lipids that was reduced when cells were treated with the TCF of LAB strains. A significant decrease in lipid levels was observed in response to treatment with 50 *μ*l of LAB TCF. Among the LABs, the TCF of LPL exhibited better activity than other strains, suggesting that the reduction of lipids by the TCF of LABs is probably because of attenuation of lipogenesis and stimulation of lipolysis and oxidation [29, 30]. We next analyzed whether the TCF of LABs attenuated LPS-induced inflammatory activity in HepG2 cells. Lipopolysaccharide is a known inducer of inflammatory response in different cell types, including HepG2 cells [25]. Treatment of cells with LPS induced the expression of exaggerated proinflammatory cytokines such as IL-6, IL-1, TGF-*β*, and TNF-*α* in vitro [24, 31]. The production of these cytokines has been shown to play critical roles in the pathogenesis of liver inflammation and liver damage. Ewaschuk et al. [19] reported that mice injected with LPS/GalN showed significant liver injury as a result of increasing permeability of the intestine and gut bacterial translocation, as well as upregulation of the production of proinflammatory cytokines in vivo.

Probiotics are promising candidates for the maintenance of human health that are able to prevent intestinal damage and reduce liver inflammation and injury [32, 33]. Oral gavages of probiotics (VSL#3) have been shown to prevent the breakdown of intestinal barrier integrity, reducing the translocation of gut bacteria and liver injury in a sepsis mouse model via peroxisome proliferator-activated receptor gamma- (PPAR*γ*-) dependent mechanisms [19]. Another study of a mouse model showed that probiotic *L. rhamnosus* GG administration attenuated hepatic steatosis and liver injury through maintenance of gut barrier functions and balance of the proportion of Th17 and Treg cell populations [34]. In addition, mice fed with probiotic MIYAIRI 588 decreased the deposition of hepatic fibrosis as well as the development of hepatocarcinogenesis and GST-P-positive foci by increasing the activation of adenosine 5-monophosphate-activated protein kinase (AMPK), protein kinase B (PKB), and the expression of lipogenesis-related proteins [17]. These studies confirmed the beneficial effects of probiotics against liver diseases; however, the results varied depending on strain type and quantity [35]. Therefore, in this study, we used hepatoma HepG2 cells to screen potent probiotic strains with immunomodulatory activity for human health. Our results showed that LPS was able to induce the production of proinflammatory cytokines such as IL-6, IL-8, MCP-1, and TNF-*α* in HepG2 cells. Similarly, Kang et al. [25] reported that treatment of HepG2 cells with LPS led to significantly upregulated IL-1*β*, IL-6, and TNF-*α* expressions. Cells stimulated with the TCF of LAB strains showed diminished LPS-induced proinflammatory cytokine expression in a time-dependent manner. At 3 h, the TCF of WCI and LPL significantly reduced the expression of IL-6, IL-8, MCP-1, and TNF-*α*, whereas the TCF of LSA failed to reduce the expression of IL-8 and MCP-1 in FFA-treated HepG2 cells. At 12 h, the mRNA level of IL-6 was reduced by the TCF of all LABs in FFA-treated cells, but the TCF of LSA did not induce a reduction in the levels of TNF-*α* in FFA-treated cells. Conversely, stimulation of FFA-untreated cells with the TCF of LABs exhibited different inhibition profiles than FFA-treated cells. Probiotic (VSL#3) administration reduced the level of hepatic IL-6 but had no effect on the level of hepatic TNF-*α* in a mouse model of sepsis [19]. IL-6 is a key inflammatory mediator of sepsis that may alter intestinal barrier function by increasing expression of the keratin gene in intestinal epithelial cells [36].

TNF-*α* is a critical proinflammatory mediator of inflammation and host immune response. Elevated levels of TNF-*α* play several crucial roles in the development of liver diseases through the upregulation of proinflammatory cytokines and the expression of lipid metabolic and fibrogenesis markers in the livers of mice [37]. TNF knockout reduced the progression of hepatic steatosis and fibrosis by downregulation of MCP-1, TGF-1*β*, Col1a1, and TIMP-1 in transgenic mice. Additionally, increases in the level of TNF-*α* were observed in the blood circulation and liver tissues of hepatic steatosis patients, which indicate that it is closely associated with disease severity and morbidity [38]. Anti-TNF-*α* antibody administration attenuated hepatic insulin resistance and progression of hepatic steatosis in a mouse model [21]. Therefore, we next analyzed whether the TCF of LABs reduces LPS-induced TNF-*α* production at the protein level in vitro. Stimulation of THP-1 cells with LPS increased the production of TNF-*α*, which was dampened by LAB treatment. The LAB LSA exhibited significantly higher reduction than other LAB strains. In addition, pretreatment of LABs upregulated the expression of IL-10 in THP-1 cells, but downregulated the level of TGF-*β*. IL-10 is a well-known anti-inflammatory cytokine that plays crucial roles in improving host immune response against pathogenic invaders and preventing inflammatory and autoimmune diseases [39]. IL-10 deficiency increased the development of chronic enterocolitis and inflammatory bowel disease in mice [40, 41] and has also been shown to protect hepatic injury in mice induced by LPS and concanavalin A. Treatment with anti-IL-10 antibody increased the severity of hepatitis and IL-12, IFN-*γ*, and TNF-*α* in the serum of mice [42].

Conversely, Smad-independent TGF-*β* signaling differentially induced the progression of hepatocellular carcinoma [43]. Administration of VSL#3 probiotics inhibited TGF-*β* signaling and its expression in mice, leading to reduced progression of MCD-induced liver fibrosis in vivo [44]. In addition, LPS treatment induced the mRNA level of IL-6, which was reduced when THP-1 cells were pretreated with all LABs except LSA. Similarly, the level of IL-1*β* was also diminished by all LABs, but not by LPL in THP-1 cells. Furthermore, LAB stimulation did not alter the level of TLR2, whereas it upregulated TLR4 expression in THP-1 cells. TLR4/LPS signaling has been reported to induce activation of MAPKs and NF-*κ*B pathways, both of which play vital and important roles in the regulation of several cellular physiological processes and host immune functions [45]. Upon recognition of LPS, TLR4 activates these pathways, which induces exacerbated forms of inflammatory states in host cells by increasing the production of proinflammatory cytokines: IL-6, IL-8, IL-1*β*, CXCL2, TNF-*α*, and others [45]. The results of our study showed that the TCFs of LABs were able to decrease the phosphorylation of p38 MAPK and p65 NF-*κ*B in HepG2 cells.

TLR4/LPS signaling is known to induce exacerbation of proinflammatory response; therefore, regulatory pathways are needed to prevent TLR4-induced inflammatory damage in the host. TLR4/LPS signaling can be regulated at different levels that may from a receptor level to transcription level via mechanisms such as deubiquitination, proteolytic degradation, and competition [46]. Some proteins have been shown to regulate the pathway of TLR4 signaling [47], and the expression of such regulatory proteins has been induced by TLR4 activation, with their increased production leading to the termination of the activation of TLR4 through negative feedback mechanisms [46]. Therefore, we investigated whether the TCFs of LABs modulate the expression of TLR-negative regulatory proteins in HepG2 cells. The results of our study showed that the TCFs of LABs were able to regulate the LPS-mediated activation of TLR signaling by modulating the expression of TLR-negative regulators such as A20, SIGIRR, Tollip, and IRAKM in HepG2 cells. The TCF of LABs diminished the expression of A20 in FFA-treated cells, whereas it upregulated Tollip, SIGIRR, and IRAKM expression in FFA-untreated cells. SIGIRR is a cell receptor that can control the activation of TLR4 signaling, and its deficiency in mice is more susceptible to LPS-induced inflammatory response [48]. IRAKM is another kind of regulatory protein that inhibits TLR4 signaling by preventing the separation of IRAKs from MyD88 protein [46]. Mice lacking IRAKM showed increased expression of inflammatory cytokines/chemokines in response to TLR4 ligands [49]. Overall, the results of this study indicated that the TCFs of LABs were able to attenuate LPS-induced inflammatory responses in vitro; however, this study has some limitations. The analysis of additional MAPKs and NF-*κ*B proteins and in vivo study helps confirming the beneficial activity of these selected strains and better understanding of molecular mechanisms behind the pathogenic and beneficial role of LPS and probiotics.

## 5. Conclusion

This study shows the immunoregulatory potential of LAB strains against bacterial LPS-induced inflammatory response in vitro. The TCFs of LABs were able to suppress HepG2 cells to produce higher levels of inflammatory cytokines such as IL-6, IL-8, MCP-1, and TNF-*α* in response to LPS. LABs achieved these beneficial effects through the modulation of TLR-negative regulators such as A20, Tollip, SIGIRR, and IRAKM, as well as the inhibition of MAPK and NF-*κ*B activation. Moreover, the TCF of LABs indirectly reduced the expression of inflammatory and anti-inflammatory cytokines in THP-1 cells. These results suggest that the TCF of LABs can attenuate inflammatory responses induced by LPS in vitro; however, a further in vivo study is needed to confirm their beneficial activity.

## Data Availability

The data used to support the findings of this study are available from the corresponding author upon request.

## Supplementary Materials

Supplementary 1 Supplementary Table 1: list of primers used for RT-PCR.

Supplementary 2 Supplementary Figure 1: western blots of phosphorylated p65 and p38 at different time periods.
