# Supplementary material for: Immunobiotics Beneficially Modulate TLR4 Signaling Triggered by Lipopolysaccharide and Reduce Hepatic Steatosis In Vitro
Source: J Immunol Res. 2019 Mar 14;2019:3876896. doi: 10.1155/2019/3876896 (PMC6437725; doi:10.1155/2019/3876896)
Supplement: Supplementary 1 — Supplementary Table 1: list of primers used for RT-PCR. [file 3876896.f1.docx]

**Suppl. Table 1** List of primers used for RT-PCR

**Target Primer sequences Annealing**

**Gene Temperature**

β-actin F5-CAAGAGATGGCCACGGCTGCT-3 63^o^C

R5-TCCTTCTGCATCCTGTCGGCA-3

IL-6 F5-CATCCTCGACGGCATCTCAG-3 63^o^C

R5-GCTCTGTTGCCTGGTCCTC-3

CXCL8 F5-CTGGCCGTGGCTCTCTTG-3 63^o^C

R5-CCTTGGCAAAACTGCACCTT-3

CCL2 F5-CTCAGCCAGATGCAATCAATG-3 63^o^C

R5-AGATCACAG CTTCTTTGGGACAC-3

A20 F5-CTG CCC AGG AAT GCT ACA GAT AC-3 63^o^C

R5-GTG GAA CAG CTC GGA TTT CAG-3

IRAKM-1 F5-AGC TGC GGG ATC TCC TTA GAG-3 63^o^C

R5-ACC GGC CTG CCA AAC AG-3

Tollip F5-CAG GCG TGG ACT CTT TCT ATC TC-3 63^o^C

R5-GAC TCC GGG ATG GTG ATG TG-3

SIGIRR F5-TTC AGT CCA GTG GCT GAA AGA CGG-3 63^o^C

R5-ACC TCT GAC AGG TTG GCC TTG AC-3

IL-10 F5-TCA GGG TGG CGA CTC TAT-3 60^o^C

R5-TGG GCT TCT TC TAA ATC GTT C-3

TLR2 F5-GCA GAA GCG CTG GGG AAT GG-3 60^o^C

R5-GGA TGC CTA CTG GGT GGA GAA-3

TLR4 F5-GGT GGA AGT TGA ACG AAT GG-3 60^o^C

R5-CCA GCA AGA AGC ATCAGG TG-3

TGF-β   F5-GCT GCT GTG GCT ACT GGT GC-3 60^o^C

R5-CAT AGA TTT CGT TGT GGG TTT C-3
