# Supplementary material for: Immunobiotics Beneficially Modulate TLR4 Signaling Triggered by Lipopolysaccharide and Reduce Hepatic Steatosis In Vitro
Source: J Immunol Res. 2019 Mar 14;2019:3876896. doi: 10.1155/2019/3876896 (PMC6437725; doi:10.1155/2019/3876896)
Supplement: Supplementary 2 — Supplementary Figure 1: western blots of phosphorylated p65 and p38 at different time periods. [file 3876896.f2.docx]

Supplementary figure

Supl. Fig. 1 Western blot of phosphorylated p65 and p38 at different time periods.
